# 4-Meth­oxy­benzamidinium nitrate

**DOI:** 10.1107/S1600536812045874

**Published:** 2012-11-10

**Authors:** Simona Irrera, Gustavo Portalone

**Affiliations:** a Chemistry Department, ‘Sapienza’ University of Rome, P.le A. Moro, 5, I-00185 Rome, Italy

## Abstract

The title salt, C_8_H_11_N_2_O^+^·NO_3_^−^, was synthesized by a reaction between 4-meth­oxy­benzamidine (4-amidino­anisole) and nitric acid. The asymmetric unit comprises a non-planar 4-meth­oxy­benzamidinium cation and a nitrate anion. In the cation, the amidinium group has two similar C—N bond lengths [1.302 (3) and 1.313 (3) Å] and its plane forms a dihedral angle of 32.66 (5)° with the mean plane of the benzene ring. The nitrate–amidinium ion pair is not planar, as the dihedral angle between the planes defined by the CN_2_^+^ and NO_3_^−^ units is 19.28 (6)°. The ionic components are associated in the crystal *via* N—H⋯O hydrogen bonds, resulting in a three-dimensional network.

## Related literature

For the biological and pharmacological relevance of benzamidine, see: Powers & Harper (1999 ▶); Grzesiak *et al.* (2000 ▶). For structural analysis of proton-transfer adducts containing mol­ecules of biological inter­est, see: Portalone (2011*a* ▶); Portalone & Irrera (2011 ▶). For the supra­molecular association in proton-transfer adducts containing benzamidinium cations, see; Portalone (2010 ▶, 2011*b* ▶, 2012 ▶); Irrera & Portalone (2012 ▶); Irrera *et al.* (2012 ▶). For hydrogen-bond motifs, see: Bernstein *et al.* (1995 ▶).
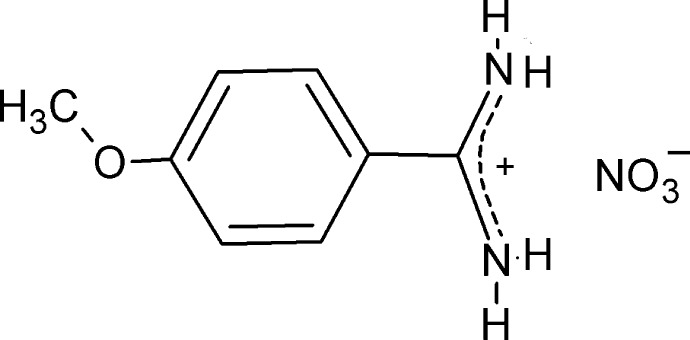


## Experimental

### 

#### Crystal data

C_8_H_11_N_2_O^+^·NO_3_^−^
*M* *_r_* = 213.20
Orthorhombic, 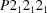

*a* = 7.1049 (7) Å
*b* = 10.3558 (8) Å
*c* = 13.4325 (9) Å
*V* = 988.32 (14) Å^3^
*Z* = 4
Mo *K*α radiation
μ = 0.12 mm^−1^
*T* = 298 K
0.20 × 0.10 × 0.08 mm

#### Data collection

Oxford Diffraction Xcalibur S CCD diffractometer
Absorption correction: multi-scan (*CrysAlis RED*; Oxford Diffraction, 2006 ▶) *T*_min_ = 0.977, *T*_max_ = 0.991
6206 measured reflections
1253 independent reflections
989 reflections with *I* > 2σ(*I*)
*R*_int_ = 0.028

#### Refinement

*R*[*F* ^2^ > 2σ(*F* ^2^)] = 0.038
*wR*(*F* ^2^) = 0.083
*S* = 0.98
1253 reflections
154 parameters
H atoms treated by a mixture of independent and constrained refinement
Δρ_max_ = 0.12 e Å^−3^
Δρ_min_ = −0.20 e Å^−3^

### 

Data collection: *CrysAlis CCD* (Oxford Diffraction, 2006 ▶); cell refinement: *CrysAlis CCD*; data reduction: *CrysAlis RED* (Oxford Diffraction, 2006 ▶); program(s) used to solve structure: *SIR97* (Altomare *et al.*, 1999 ▶); program(s) used to refine structure: *SHELXL97* (Sheldrick, 2008 ▶); molecular graphics: *ORTEP-3* (Farrugia, 2012 ▶); software used to prepare material for publication: *WinGX* (Farrugia, 2012 ▶).

## Supplementary Material

Crystal structure: contains datablock(s) global, I. DOI: 10.1107/S1600536812045874/fi2127sup1.cif

Structure factors: contains datablock(s) I. DOI: 10.1107/S1600536812045874/fi2127Isup2.hkl

Additional supplementary materials:  crystallographic information; 3D view; checkCIF report

## Figures and Tables

**Table 1:** Hydrogen-bond geometry (Å, °)

*D*—H⋯*A*	*D*—H	H⋯*A*	*D*⋯*A*	*D*—H⋯*A*
N1—H1*A*⋯O1	0.87 (2)	2.00 (2)	2.850 (3)	166 (2)
N1—H1*B*⋯O3^i^	0.91 (2)	2.10 (2)	3.008 (3)	170 (2)
N2—H2*A*⋯O2	0.87 (2)	2.05 (2)	2.920 (3)	175 (2)
N2—H2*B*⋯O1^ii^	0.84 (3)	2.43 (3)	3.030 (3)	129 (2)
N2—H2*B*⋯O3^ii^	0.84 (3)	2.33 (3)	3.174 (3)	177 (2)

Symmetry codes: (i) 
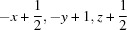
; (ii) 
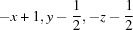
.

**Fig. 1.**
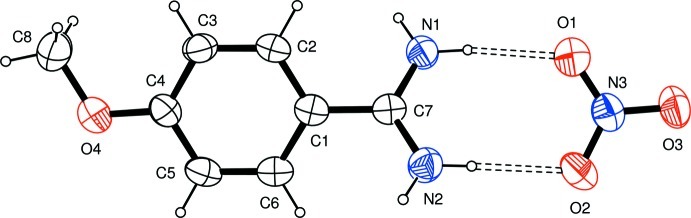
The asymmetric unit of the title compound. Displacements ellipsoids are at the 50% probability level. The asymmetric unit was selected so that the two ions are linked by N+.—H···O- hydrogen bonds. H atoms are shown as small spheres of arbitrary radii. Hydrogen bonds are indicated by dashed lines.

**Fig. 2.**
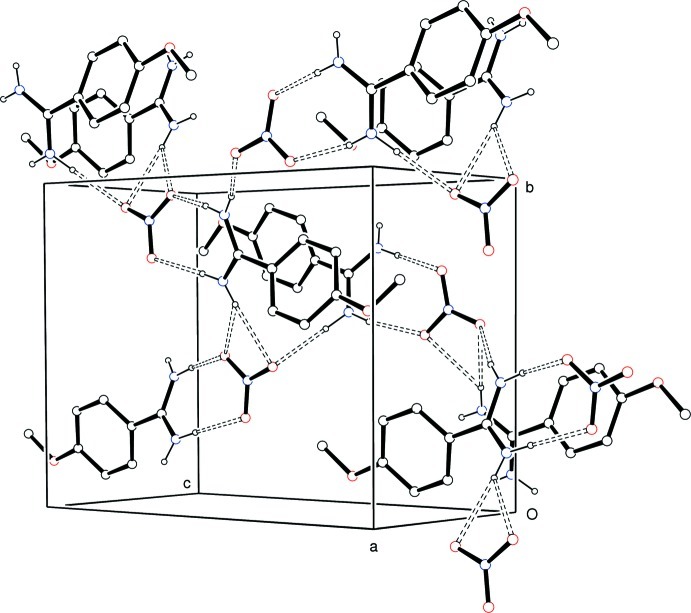
Crystal packing diagram for the title compound. All atoms are shown as small spheres of arbitrary radii. For the sake of clarity, H atoms not involved in hydrogen bonding have been omitted. Hydrogen bonding is indicated by dashed lines.

## supplementary crystallographic information

### Comment

Following our on-going interest on systematic structural analysis of proton-transfer adducts containing molecules of biological interest (Portalone, 2011*a*; Portalone & Irrera, 2011), benzamidine derivatives, which have shown strong biological and pharmacological activity (Powers & Harper, 1999; Grzesiak *et al.*, 2000), are being used in our group as bricks for supramolecular construction (Portalone, 2010, 2011*b*, 2012). Indeed, these molecules are strong Lewis bases and their cations can be easily anchored onto numerous inorganic and organic anions and polyanions, largely because of the presence of four potential donor sites for hydrogen-bonding.

We report here the crystal structure of the title compound, 4-methoxybenzamidinium nitrate, which was obtained by a reaction between 4-methoxybenzamidine (4-amidinoanisole) and nitric acid.

The asymmetric unit of the title compound comprises one non-planar 4-methoxybenzamidinium cation and one nitrate anion (Fig. 1).

In the cation, the amidinium group forms dihedral angle of 32.66 (5)° with the mean plane of the phenyl ring, which is close to the the values observed in protonated benzamidinium ions (14.4 (1) - 30.4 (1)°, Portalone, 2010, 2012; Irrera *et al.*, 2012). The lack of planarity in all these systems is obviously caused by steric hindrances between the H atoms of the aromatic ring and the amidine group. This conformation is rather common in benzamidinium-containing small-molecule crystal structures, with the only exception of benzamidinium diliturate, where the benzamidinium cation is planar (Portalone, 2010). The pattern of bond lengths and bond angles of the 4-methoxybenzamidinium cation agrees with that reported in previous structural investigations (Irrera *et al.*, 2012; Portalone, 2010, 2012; Irrera & Portalone, 2012). In particular the amidinium group, true to one's expectations, features similar C—N bonds [1.302 (3) and 1.313 (3) Å], evidencing the delocalization of the π electrons and double-bond character. The molecular parameters for the nitrate ion are in the expected range.

The ionic components of compound (I) are joined by two N^+^—H···O^-^ (±) hydrogen bonds to form ionic dimers with graph-set motif *R*^2^_2_(8) (Bernstein *et al.*, 1995). Remarkably, at variance with the well known carboxylic dimer *R*^2^_2_ (8) motif, the nitrate-amidinium pair is not planar, as the dihedral angles for the planes defined by the CN_2_^+^ and NO_3_^-^ atoms is 19.28 (6)°. Similar deviation from planarity has been previously observed in the carboxylate-amidinium pair of benzamidinium 2,6-dimethoxybenzoate (Irrera *et al.*, 2012).

Analysis of the crystal packing of the title compound (Fig. 2) shows that each amidinium unit is bound to three nitrate anions by five distinct N—H^+^···O^-^ intermolecular hydrogen bonds (N^+^···O^-^ = 2.850 (3) - 3.174 (3) Å, Table 1). Four cation-to-anion N^+^···O^-^ interactions form two types of hydrogen-bonded rings: the *R*^2^_1_(4), which indicates a bifurcated hydrogen bond from N2—H2b to the two acceptors (O1, O3), and the already mentioned *R*^2^_2_(8) supramolecular synthon. Both these ring motifs lead to the formation of ribbons approximately along crystallographic *b* axis. These ribbons are then interconnected by means of the remaining N^+^···O^-^ intermolecular hydrogen bond.

### Experimental

4-methoxybenzamidine (0.1 mmol, Fluka at 96% purity) was dissolved without further purification in 6 ml of hot water and heated under reflux for 3 h. While stirring, HNO_3_ (4 mol *L*^-1^) was added dropwise until pH reached 2. After cooling the solution to an ambient temperature, colourless crystals suitable for single-crystal X-ray diffraction were grown by slow evaporation of the solvent after one week.

### Refinement

All H atoms were identified in difference Fourier maps, but for refinement all C-bound H atoms were placed in calculated positions, with C—H = 0.93 Å (phenyl) and 0.96 Å (methyl), and refined as riding on their carrier atoms. The *U*_iso_ values were kept equal to 1.2*U*_eq_(C, phenyl). and to 1.5*U*_eq_(C, methyl). Positional and thermal parameters of H atoms of the amidinium group were freely refined, giving N—H distances in the range 0.84 (3) - 0.91 (2) Å. In the absence of significant anomalous scattering in this light-atom study of the title compound, Friedel pairs were merged prior to refinement.

### Crystal data

**Table:**

C_8_H_11_N_2_O^+^·NO_3_^−^	*F*(000) = 448
*M**_r_* = 213.20	*D*_x_ = 1.433 Mg m^−^^3^
Orthorhombic, *P*2_1_2_1_2_1_	Mo *K*α radiation, λ = 0.71069 Å
Hall symbol: P 2ac 2ab	Cell parameters from 2927 reflections
*a* = 7.1049 (7) Å	θ = 3.0–29.0°
*b* = 10.3558 (8) Å	µ = 0.12 mm^−^^1^
*c* = 13.4325 (9) Å	*T* = 298 K
*V* = 988.32 (14) Å^3^	Tablets, colourless
*Z* = 4	0.20 × 0.10 × 0.08 mm

### Data collection

**Table:**

Oxford Diffraction Xcalibur S CCD diffractometer	1253 independent reflections
Radiation source: Enhance (Mo) X-ray Source	989 reflections with *I* > 2σ(*I*)
Graphite monochromator	*R*_int_ = 0.028
Detector resolution: 16.0696 pixels mm^-1^	θ_max_ = 27.0°, θ_min_ = 3.0°
ω and φ scans	*h* = −8→8
Absorption correction: multi-scan (*CrysAlis RED*; Oxford Diffraction, 2006)	*k* = −13→12
*T*_min_ = 0.977, *T*_max_ = 0.991	*l* = −17→17
6206 measured reflections	

### Refinement

**Table:**

Refinement on *F*^2^	Primary atom site location: structure-invariant direct methods
Least-squares matrix: full	Secondary atom site location: difference Fourier map
*R*[*F*^2^ > 2σ(*F*^2^)] = 0.038	Hydrogen site location: inferred from neighbouring sites
*wR*(*F*^2^) = 0.083	H atoms treated by a mixture of independent and constrained refinement
*S* = 0.98	*w* = 1/[σ^2^(*F*_o_^2^) + (0.0527*P*)^2^ + 0.0001*P*] where *P* = (*F*_o_^2^ + 2*F*_c_^2^)/3
1253 reflections	(Δ/σ)_max_ < 0.001
154 parameters	Δρ_max_ = 0.12 e Å^−^^3^
0 restraints	Δρ_min_ = −0.20 e Å^−^^3^

### Special details

**Table:**

Geometry. All s.u.'s (except the s.u. in the dihedral angle between two l.s. planes) are estimated using the full covariance matrix. The cell s.u.'s are taken into account individually in the estimation of s.u.'s in distances, angles and torsion angles; correlations between s.u.'s in cell parameters are only used when they are defined by crystal symmetry. An approximate (isotropic) treatment of cell s.u.'s is used for estimating s.u.'s involving l.s. planes.
Refinement. Refinement of *F*^2^ against ALL reflections. The weighted *R*-factor *wR* and goodness of fit *S* are based on *F*^2^, conventional *R*-factors *R* are based on *F*, with *F* set to zero for negative *F*^2^. The threshold expression of *F*^2^ > 2σ(*F*^2^) is used only for calculating *R*-factors(gt) *etc*. and is not relevant to the choice of reflections for refinement. *R*-factors based on *F*^2^ are statistically about twice as large as those based on *F*, and *R*- factors based on ALL data will be even larger.

### Fractional atomic coordinates and isotropic or equivalent isotropic displacement parameters (Å^2^)

**Table:**

	*x*	*y*	*z*	*U*_iso_*/*U*_eq_	
O4	0.3500 (3)	0.10530 (16)	0.33730 (12)	0.0565 (5)	
N1	0.3218 (3)	0.3966 (2)	−0.07851 (16)	0.0441 (5)	
H1A	0.325 (3)	0.426 (2)	−0.1390 (18)	0.041 (6)*	
H1B	0.268 (3)	0.448 (2)	−0.0308 (18)	0.044 (7)*	
N2	0.4153 (3)	0.1984 (2)	−0.12938 (16)	0.0454 (5)	
H2A	0.408 (3)	0.222 (3)	−0.1914 (18)	0.046 (7)*	
H2B	0.463 (3)	0.127 (3)	−0.1138 (17)	0.045 (8)*	
C1	0.3682 (3)	0.2331 (2)	0.04594 (15)	0.0359 (5)	
C2	0.4110 (3)	0.3182 (2)	0.12224 (14)	0.0377 (5)	
H2	0.4426	0.4032	0.1072	0.045*	
C3	0.4073 (3)	0.2785 (2)	0.22025 (16)	0.0419 (5)	
H3	0.4372	0.3363	0.2708	0.050*	
C4	0.3588 (3)	0.1520 (2)	0.24330 (16)	0.0397 (5)	
C5	0.3158 (3)	0.0660 (2)	0.16755 (17)	0.0432 (5)	
H5	0.2832	−0.0188	0.1827	0.052*	
C6	0.3215 (3)	0.1061 (2)	0.07021 (17)	0.0401 (5)	
H6	0.2937	0.0478	0.0197	0.048*	
C7	0.3686 (3)	0.2769 (2)	−0.05791 (15)	0.0347 (5)	
C8	0.3767 (4)	0.1947 (3)	0.41712 (18)	0.0639 (8)	
H8A	0.498 (3)	0.2338 (16)	0.4114 (9)	0.096*	
H8B	0.368 (3)	0.1501 (8)	0.4793 (11)	0.096*	
H8C	0.282 (2)	0.2602 (17)	0.4141 (9)	0.096*	
N3	0.3829 (3)	0.3901 (2)	−0.35182 (15)	0.0471 (5)	
O1	0.3880 (3)	0.46641 (18)	−0.28077 (14)	0.0669 (5)	
O2	0.3696 (3)	0.27255 (18)	−0.33771 (13)	0.0735 (6)	
O3	0.3962 (3)	0.43439 (17)	−0.43752 (12)	0.0611 (5)	

### Atomic displacement parameters (Å^2^)

**Table:**

	*U*^11^	*U*^22^	*U*^33^	*U*^12^	*U*^13^	*U*^23^
O4	0.0789 (12)	0.0439 (10)	0.0466 (9)	−0.0041 (10)	0.0002 (8)	0.0075 (9)
N1	0.0525 (12)	0.0399 (12)	0.0401 (11)	0.0049 (10)	0.0049 (9)	0.0023 (10)
N2	0.0481 (12)	0.0456 (14)	0.0425 (12)	0.0050 (11)	0.0003 (9)	−0.0013 (10)
C1	0.0272 (10)	0.0357 (12)	0.0448 (11)	0.0026 (9)	0.0010 (9)	−0.0008 (10)
C2	0.0386 (12)	0.0279 (12)	0.0466 (12)	−0.0024 (9)	−0.0005 (9)	−0.0001 (10)
C3	0.0477 (13)	0.0346 (11)	0.0433 (11)	−0.0016 (11)	−0.0055 (10)	−0.0071 (11)
C4	0.0403 (12)	0.0363 (12)	0.0426 (11)	0.0034 (10)	0.0002 (9)	0.0048 (11)
C5	0.0460 (13)	0.0297 (12)	0.0537 (13)	−0.0028 (11)	0.0043 (11)	0.0007 (11)
C6	0.0370 (11)	0.0345 (12)	0.0488 (12)	−0.0006 (10)	−0.0001 (10)	−0.0055 (11)
C7	0.0264 (10)	0.0359 (12)	0.0416 (11)	0.0001 (9)	0.0020 (9)	−0.0032 (11)
C8	0.0867 (19)	0.0635 (18)	0.0415 (12)	−0.0123 (17)	−0.0007 (13)	0.0001 (13)
N3	0.0480 (11)	0.0489 (12)	0.0446 (10)	0.0027 (10)	−0.0002 (10)	0.0090 (11)
O1	0.0978 (14)	0.0531 (11)	0.0498 (9)	0.0019 (11)	0.0058 (10)	0.0028 (9)
O2	0.1182 (17)	0.0438 (11)	0.0586 (10)	−0.0156 (12)	−0.0060 (12)	0.0124 (9)
O3	0.0836 (12)	0.0565 (11)	0.0434 (9)	0.0070 (10)	0.0062 (9)	0.0164 (9)

### Geometric parameters (Å, º)

**Table:**

O4—C4	1.354 (3)	C3—C4	1.390 (3)
O4—C8	1.429 (3)	C3—H3	0.9300
N1—C7	1.313 (3)	C4—C5	1.387 (3)
N1—H1A	0.87 (2)	C5—C6	1.373 (3)
N1—H1B	0.91 (2)	C5—H5	0.9300
N2—C7	1.302 (3)	C6—H6	0.9300
N2—H2A	0.87 (2)	C8—H8A	0.9572
N2—H2B	0.84 (3)	C8—H8B	0.9572
C1—C2	1.386 (3)	C8—H8C	0.9572
C1—C6	1.395 (3)	N3—O2	1.235 (3)
C1—C7	1.467 (3)	N3—O1	1.240 (3)
C2—C3	1.379 (3)	N3—O3	1.243 (2)
C2—H2	0.9300		
			
C4—O4—C8	117.53 (18)	C6—C5—C4	119.9 (2)
C7—N1—H1A	121.4 (16)	C6—C5—H5	120.1
C7—N1—H1B	120.3 (14)	C4—C5—H5	120.1
H1A—N1—H1B	118 (2)	C5—C6—C1	121.0 (2)
C7—N2—H2A	120.8 (17)	C5—C6—H6	119.5
C7—N2—H2B	118.0 (16)	C1—C6—H6	119.5
H2A—N2—H2B	121 (2)	N2—C7—N1	120.0 (2)
C2—C1—C6	118.62 (19)	N2—C7—C1	120.6 (2)
C2—C1—C7	120.41 (18)	N1—C7—C1	119.49 (19)
C6—C1—C7	120.95 (19)	O4—C8—H8A	109.5
C3—C2—C1	120.8 (2)	O4—C8—H8B	109.5
C3—C2—H2	119.6	H8A—C8—H8B	109.5
C1—C2—H2	119.6	O4—C8—H8C	109.5
C2—C3—C4	119.9 (2)	H8A—C8—H8C	109.5
C2—C3—H3	120.1	H8B—C8—H8C	109.5
C4—C3—H3	120.1	O2—N3—O1	120.82 (19)
O4—C4—C5	116.40 (19)	O2—N3—O3	120.8 (2)
O4—C4—C3	123.8 (2)	O1—N3—O3	118.4 (2)
C5—C4—C3	119.8 (2)		
			
C6—C1—C2—C3	−0.1 (3)	C3—C4—C5—C6	0.1 (3)
C7—C1—C2—C3	178.67 (18)	C4—C5—C6—C1	−0.6 (3)
C1—C2—C3—C4	−0.5 (3)	C2—C1—C6—C5	0.6 (3)
C8—O4—C4—C5	−174.4 (2)	C7—C1—C6—C5	−178.10 (19)
C8—O4—C4—C3	5.5 (3)	C2—C1—C7—N2	148.0 (2)
C2—C3—C4—O4	−179.38 (19)	C6—C1—C7—N2	−33.3 (3)
C2—C3—C4—C5	0.5 (3)	C2—C1—C7—N1	−31.7 (3)
O4—C4—C5—C6	179.9 (2)	C6—C1—C7—N1	147.0 (2)

### Hydrogen-bond geometry (Å, º)

**Table:**

*D*—H···*A*	*D*—H	H···*A*	*D*···*A*	*D*—H···*A*
N1—H1*A*···O1	0.87 (2)	2.00 (2)	2.850 (3)	166 (2)
N1—H1*B*···O3^i^	0.91 (2)	2.10 (2)	3.008 (3)	170 (2)
N2—H2*A*···O2	0.87 (2)	2.05 (2)	2.920 (3)	175 (2)
N2—H2*B*···O1^ii^	0.84 (3)	2.43 (3)	3.030 (3)	129 (2)
N2—H2*B*···O3^ii^	0.84 (3)	2.33 (3)	3.174 (3)	177 (2)

Symmetry codes: (i) −*x*+1/2, −*y*+1, *z*+1/2; (ii) −*x*+1, *y*−1/2, −*z*−1/2.
